# Prevalence and predictors of hepatitis C infection among antenatal attendees in a tertiary hospital in Southern Nigeria

**DOI:** 10.4314/ahs.v23i3.8

**Published:** 2023-09

**Authors:** Ganiyu Oluwedolapo Shittu, Aniekan Monday Abasiattai, Aniefiok Jackson Umoiyoho, Ifeanyi Abraham Onwuezobe

**Affiliations:** 1 Department of Obstetrics/Gynaecology, University of Uyo Teaching hospital, Uyo, Nigeria; 2 Department of Microbiology and Parasitology, University of Uyo Teaching Hospital, Uyo, Nigeria

**Keywords:** Hepatitis C virus infection, pregnant women, Uyo, prevalence of hepatitis C

## Abstract

**Background:**

Hepatitis C virus infection as it specifically relates to pregnancy has been a neglected condition, thus its recognition and treatment in pregnancy is relevant because of the risks of the long-term complications of the infection in the mother, potential effects of the infection on the pregnancy and risk of vertical transmission to the newborn.

**Objectives:**

To determine the proportion of pregnant women with serologic markers of hepatitis C infection, identify risk factors as well as factors that predict the occurrence of the infection in them.

**Methodology:**

Over a 3-week period, blood samples from 456 pregnant women were assessed for antibodies to hepatitis C virus, while a pre-tested questionnaire was used to obtain socio-demographic data and the presence of risk factors in the University of Uyo Teaching Hospital, Nigeria.

**Results:**

The prevalence of HCV infection in pregnancy was 4.6%. No known risk factors for HCV infection in pregnancy were identified. Only increasing gestational age was a predictor of HCV infection in pregnancy in the study.

**Conclusion:**

The prevalence of hepatitis virus infection among the study population was high. Second trimester and increasing gravidity were protective of the infection in pregnancy. There is therefore need for introduction of general routine screening of all pregnant women presenting for antenatal care.

## Introduction

Hepatitis C virus (HCV), a blood borne pathogen discovered nearly three decades ago is a major cause of chronic liver disease worldwide and the leading indication for liver transplantation in the western world.^1^ It is estimated that 130-200 million people or 3% of the world's population are living with chronic hepatitis C.^2^ Over 80% of these chronically infected people are asymptomatic and 55-85% progress to chronic liver disease in a slow and insidious manner^3^.

The prevalence of HCV infection varies in different parts of the world. It is less than 0.5% in the Scandinavian countries, 3.2% in China, 4.8% in Pakistan and 22% in Egypt.^2^ Although there are no country wide surveys on the epidemiology of HCV in Nigeria, the prevalence of HCV infection appears to be rising 4.

Hepatitis C virus transmission occurs principally through parenteral exposure to contaminated blood or blood products in adults 5 as well as through mother to child transmission in children with a transmission rate of 6%.^6^

The majority of vertical HCV transmission occurs at the time of delivery^7^. Early studies suggest that transmission is more common when maternal viral load is high or in the presence of concomitant HIV infection^8^,^9^. Breast-feeding is not thought to increase infection risk to babies^7^. Hepatitis C virus is not found consistently in body secretions, thus sexual transmission is rare^10^. Other risk factors for HCV infection include acupuncture, tattooing, haemodialysis, occupational exposure in health-care workers and the re-use of needles in mass vaccination programs^5^.

A small proportion of patients with no identifiable risk factor are designated as sporadic cases.

Pregnant women are among groups less often discussed and studied when considering the burden of HCV infection. In pregnant populations, advances in HCV science, therapies, and policies for adults have not been matched even though HCV mono-infected pregnant women are known to have a high risk of viral transmission to their babies.

Recognition of HCV infection in pregnancy is relevant because of the risks of the long-term complications of the infection in the mother (Liver Cirrhosis and Hepatocellular Carcinoma), potential effects of the infection on the pregnancy and risk of vertical transmission to the newborn and household.

Antenatal HCV screening will help identify asymptomatic pregnant women with infection, identify those who may benefit from antiviral therapy and postpartum monitoring and interventions for infected mothers and children. Before now, routine antenatal HCV screening was not done for pregnant women who did not have known risk factors for infection, the main reason being the lack of effective treatment options to avoid MTCT during pregnancy or delivery. Treatment regimens based on interferon (IFN) and ribavirin (RBV) that were available, were associated with occasional severe side-effects and thus their indications had to be carefully evaluated. In addition, ribavirin is known to have teratogenic and embryocidal effects and is absolutely contraindicated in pregnancy.

This situation has significantly changed with the introduction of very effective, well tolerated, direct-acting antiviral agents (DAAs) which are newly available treatment regimens. Today, it is of advantage to know a pregnant woman's HCV status so that those infected can be referred for treatment after delivery and their neonates closely followed up to exclude vertical transmission. Importantly, HCV eradication cn be achieved without using Ribavirin. Therefore, with the new DAA regimens, clearing HCV infection before subsequent pregnancies becomes a realistic strategy to completely eradicate HCV vertical transmission in the future and routine antenatal HCV screening has been shown to be cost effective^11^.

There has been no study at the University of Uyo Teaching Hospital (UUTH) documenting the prevalence of Hepatitis C infection among pregnant women. This study will bridge this gap in knowledge and will thus assess the anti-HCV sero-prevalence in pregnant women in the centre and subsequently provide recommendations on the rationale for possible routine screening for HCV infection in our pregnant population.

## Materials and methods

### Study area

The study was carried out at the Obstetrics and Gynaecology department of UUTH, Uyo, Akwa Ibom State. The hospital, with a 400-bed capacity, is the only public tertiary health facility in the state and provides research, training and specialized health services to indigenes of the state and beyond.

### Study design

This study used a descriptive cross-sectional design to achieve the objectives of the study.

### Study population

The study population was drawn from pregnant women on their first antenatal visit during the study period. About 966 women registered for antenatal care over the three-month study period.

### Recruitment criteria

**Inclusion criteria:** All women who consented to participate in the study.

**Exclusion criteria:** Pregnant women who were too ill and also those who declined to participate.

### Sample Size determination

The sample size was determined using the formula^12^


$$\begin{array}{l} {\text{n}}^{\text{0}}={\text{Z}}^{\text{2}}\text{pq}\\ \;\;{\text{d}}^{\text{2}} \end{array}$$

Where n^0^ = minimum sample size required

Z = 1.96 (A value of standard deviation corresponding to 1.96)

P = prevalence rate from a similar study in Benin City in the same zone as Uyo = 5% 13

q = (1 − p) = 0.95

d = degree of accuracy desired, 2.0% = 0.02

Therefore $\begin{array}{l} {\text{n}}^{\text{0}}={\left(1.96\right)}^{2}\times 0.05\times 0.95\\ \;\;\;\;\;\;{\left(0.02\right)}^{2}\\ \;\;=\;3.8416\times 0.05\times 0.95\\ \;\;\;\;\;0.0004 \end{array}$

n^0^= 456.19

Allowing for 5% non-response rate =22.8

This is approximately 456.19 + 22.8 = 478.9

The minimum sample size required for the study was 480

### Sampling technique

The total population of pregnant women register for antenatal care during the period under study (3 months, 12 weeks) was approximately 972 (i.e., 81 ×12 weeks). This was the estimated sampling frame (N). The minimum sample size, n was 480. The systematic sampling technique was used to recruit respondents that meet the inclusion criteria. Every k^th^ number from the estimated sample frame was selected every week on Wednesdays during the booking clinic, where “K” is the sampling interval.

**K**= N/n

**N** = total population expected during the study period

**n** = proposed sample size

**K** = 972/ 480 = 2.02 = 2

Sampling interval was therefore approximated to 2

Women booking for antenatal care every Wednesday usually write their names on a sheet of paper in the clinic as they arrive and this list formed our sampling frame.

The starting number was selected by simple random technique using the balloting method. Thereafter, every second client was recruited into the study until the minimum sample size was obtained. For every booking clinic, about 40 clients were recruited.

### Study instrument

Data was collected using pre-tested, structured, interviewer and self-administered questionnaires which containing information on the socio-demographic and obstetric characteristics of respondents, awareness and knowledge of transmission, and signs of Hepatitis C infection as well as risk factors for infection.

### Ethical considerations

Ethical approval was sought from the Ethical Review Committee of the University of Uyo Teaching hospital, Uyo before the commencement of the study. Also, an informed consent was obtained from each respondent before the questionnaire was administered and samples taken. Participation in the study was voluntary and those who consented to participate were informed of their right to opt out at any time if they so desired, while also being assured of the confidentiality of any information obtained from them in the course of the research. Health education on ways of preventing Hepatitis C infection was given to each respondent and respondents were also informed of their hepatitis C status. Those with positive hepatitis C status were appropriately referred to the Hematology Department of the same hospital for expert management.

### Data management

Data collected was collated, cleansed and analysed using Statistical Package for Social Sciences (SPSS), version 20.0 (SPSS Inc. Chicago, IL, USA). Descriptive statistics was done for continuous variables while categorical variables were compared using Chi-Square. Level of significance was set at 5% (p<0.05). Logistic regression was performed to determine the predictors of Hepatitis C infection among respondents. Questions on knowledge of the transmission of Hepatitis C were scored with 2 marks allotted to each correct response and 1 mark for an incorrect response. Levels of knowledge were categorized as poor for knowledge scores of less than 50%, and good for scores of 50 to 100%.

### Specimen collection and transportation

All respondents recruited for the study were administered with the study questionnaire to fill (Self/interviewer-administered questionnaire). In addition, their blood samples were collected by a Laboratory Scientist, a research assistant who had been properly trained on the objectives of the study. The assay for Hepatitis C virus was performed in the Microbiology laboratory of the Teaching hospital. The test kit used was the ABON® Hepatitis C Virus antibody test device which utilizes an immune-chro-matographic technology and a high specificity of antibody-antigen reaction for the detection of antibodies to HCV in serum or plasma. It has a sensitivity of 98.75% and specificity of 99.38%.

## Results

Four hundred and eighty-five participants were recruited and complete results were obtained from 480 of them giving a response rate of 98.9%. Twenty-two patients had serological markers for HCV resulting in a prevalence of 4.6%

The ages of respondents ranged from 17 to 42 years with a mean age of 30.02 + 4.30 years. The majority 410 (85.4%) were less than 35 years. Almost all respondents (97.7%) were married and 356 (74.2%) had tertiary level of education. About half of the respondents 236 (49.2%) were in the third trimester of pregnancy. Most of them (82.1%) were between Para 1-3 and 67.7% were either nulliparous or primiparous (Table 1).

**Table 1:** Socio-demographic and obstetric characteristics of respondents

Characteristics	Frequency	Percent
**Age Group (in years)**		
Less than 35	410	85.4
35 and above	70	14.6
**Marital status**		
Single	10	2.1
Married	469	97.7
Widowed	1	0.2
**Level of Education**		
No formal education	6	1.3
Primary education	21	4.4
Secondary education	97	20.2
Tertiary education	356	74.2
**Gestational Age (in months)**		
1-3	67	14.0
4-6	177	36.9
7-9	236	49.2
**Gravidity**		
1-3	394	82.1
4 and above	86	17.9
**Parity Groups**		
0-1	325	67.7
2 and above	155	32.3
**State of origin**		
Indigenes	375	78.1
Non-Indigenes	105	21.9

Majority of the respondents (58.1%) were aware of HCV Infection while 41.9% were not.

Table 2 shows the risk factors for HCV among the respondents. The common risk factors were: not having personal hair making items (41.5%), history of blood transfusion and sexually transmitted diseases (13.9% respectively) and previous history of jaundice (13.5%).

**Table 2:** Risk factors for HCV infection among respondents (only positive responses)

Risk Factors	Frequency	Percent
Had history of yellowness of the eyes	65	13.54
Had acute or chronic liver disease	39	8.14
Have tattoo or body piercing	33	6.92
Rejection as blood donor	11	2.29
Do not Have personal combs/needles for making hair	199	41.46
Had blood transfusion in the past	67	13.96
Share tooth brush with others	41	8.56
Had STD before	67	13.96
Have regular intravenous drugs	35	7.29
Spouse had been rejected as blood donor	16	3.49
HIV positive (n=437)	25	5.72

Multiple Responses allowed.

There was an association between gestational age and Hepatitis C status of respondents (p=0.04). There was no statistically significant association between the selected characteristics and the hepatitis status of the respondents. Only the gestational age of the patient had a strong association with the HCV infection status. The Hepatitis C Virus infection status was independent of the age group (P =0.756), marital status (P = 0.593), state of origin (P= 0.797), level0.481) of the respondents (Table 3). The association between known risk factors in the respondents and their HCV status was not significant statistically (table 4).

**Table 3:** Association between socio demographic/obstetric characteristics and hepatitis C status of respondents

Characteristics	Hepatitis C status		Total	Statistical test and values
	Reactive	Non reactive		
**Age group**				x^2^ =0.558
less than 35	20 (4.9)	390 (95.1)	410	p=0.756*
35 years and above	2 (2.9)	68 (97.1)	70	df=1
**Marital status**				
single	0 (0.0)	10 (100.0)	10	x^2^ =0.541
married	22 (4.7)	447 (95.3)	469	p=0.593*
widowed	0 (0.0)	1 (100.0)	1	df=2
**State of origin**				x^2^ =0.184
indigenes	18 (4.8)	357 (95.2)	375	p=0.797f*
non indigenes	4 (3.8)	101 (96.2)	105	df=1
**Level of education**				
no formal education	1 (16.7)	5 (83.3)	6	x^2^ =3.651
primary education	1 (4.7)	20 (95.2)	21	p=0.514*
secondary education	2 (2.1)	95 (97.9)	97	df=4
tertiary education	18 (5.1)	338 (94.9)	356	
**Gestational age**				x^2^ =5.629
1-3 months	5 (7.5)	62 (92.5)	67	p=0.04 (lr)
4-6 months	3 (1.7)	174 (98.3)	177	df=2
7-9 months	14 (5.9)	222 (94.1)	236	
**Gravidity**				x^2^ =1.221
1-3	20 (5.1)	374 (94.9)	394	p=0.396*
4 and above	2 (2.3)	84 (97.7)	86	df=1
**Parity group**				x^2^ =0.965
0-1	17 (5.2)	308 (94.8)	325	p=0.481*
2 and above	5 (3.2)	150 (96.8)	155	df=1

* **Fisher Exact. LR Likelihood ratio**.

**Table 4:** Association between risk factors of respondents and their hepatitis C status

Characteristics	Hepatitis C status		Total	Statistical test and values
	Reactive	Non-reactive		
**Ever jaundiced**				X^2^ =0.390
Yes	2 (3.1)	63 (96.9)	65	P=0.753*
No	20 (4.8)	395 (95.2)	415	DF=1
**Ever had acute or chronic liver**				X^2^=0.396
**disease**				P=0.999*
Yes	1 (2.6)	38 (97.4)	39	DF=1
No	21 (4.8)	420 (95.2)	441	
**Ever had tattoo or body piercing**				X^2^ =0.195
Yes	1 (3.0)	32 (97.0)	33	P=0.999*
No	21 (4.7)	426 (95.3)	447	DF=1
**Ever been rejected as blood**				X^2^ =0.523
**donor**				P=0.406*
Yes	1 (9.1)	10 (90.9)	11	DF=1
No	21 (4.5)	448 (95.5)	469	
**Own Personal combs/hair**				X^2^ =0.883
**making needles**				P=0.385*
Yes	15 (5.3)	266 (94.7)	281	DF=1
No	7 (3.5)	192 (96.5)	199	
**Ever been transfused**				X^2^ =0.342
Yes	4 (6.0)	63 (94.0)	67	P=0.530*
No	18 (4.4)	395 (95.6)	413	DF=1
**Ever Had STD**				X^2^ =0.342
Yes	4 (6.0)	63 (94.0)	67	P=0.530*
No	18 (4.4)	395 (95.6)	413	DF=1
**Regular IV drugs**				X^2^ =0.110
Yes	2 (5.7)	33 (94.3)	35	P=0.670*
No	20 (4.5)	425 (95.5)	445	DF=1
**Share toothbrush**				X^2^ =0.471
Yes	1 (2.4)	40 (97.6)	41	P=0.710*
No	21 (4.8)	418 (95.2)	439	DF=1
**Spouse ever been rejected as**				X^2^ =2.372
**blood donor**				P=0.163*
Yes	2 (12.5)	14 (87.5)	16	DF=1
No	20 (4.3)	444 (95.7)	464	
**HIV Status (n=439)**				X^2^ =0.019
Negative	19 (4.6)	395 (95.4)	414	P=0.999*
Positive	1 (4.0)	24 (96.0)	25	DF=1

* **Fisher Exact**

Table 5 shows possible predictors of Hepatitis C virus infection among respondents. The older patient was, the less likely the chance of being HCV reactive both at the univariate and multivariate logistic regression (OR 0.92 and 0.50 respectively). However, this relationship was not statistically significant (P value: 0.12, 0.50). Though those who had no formal education and those had primary education were 1.8 times more likely to be hepatitis C reactive at the univariate logistic regression level (OR: 1.81), at the multivariate logistic regression level, having no or primary education was protective against having HCV infection (OR: 0.96). However, this relationship (education and chance of having HCV infection) was not statistically significant (P: 0.44, 0.96). Increasing gestational age was protective against HCV infection in pregnancy at both univariate (OR: 0.21(T2); 0.78(T3) and multivariate regression levels (OR: 0.18(T2); 0.58(T3). However, this relationship was only statistically significant in the second trimester (P:0.04, 0.03 for univariate and multivariate logistic regression levels respectively). Moreso, increasing gravidity also protected against HCV infection in preganancy (OR: 0.44 and 0.38, at both univariate and multivariate logistic regression levels respectively). However, this was only slightly/Statistically significant (P=0.049) at multivariate logistic regression level.

**Table 5:** Predictors of hepatitis C infection among respondents

Factors	Univariate logistic regression			Multivariate logistic regression		
	Crude	P value	95%CI	Adjusted	P value	95%CI
	OR			OR		
**Age**	0.92	0.12	0.835-1.021	0.95	0.50	0.834-1.093
**Education**						
Sec/Tertiary	1					
None/Primary	1.81	0.44	0.399-8.190	0.94	0.96	0.104-8.622
**Gestational Age**						
1^st^ Trimester	1					
2^nd^ Trimester	0.21	0.038	0.049-0.920	0.18	0.03*	0.038-0.837
3^rd^ Trimester	0.78	0.649	0.271-2.255	0.58	0.36	0.182-1.840
**Gravidity**	0.44	0.28	0.102-1.941	0.38	0.049*	0.146-0.994
**Parity**						
0-1	1					
2 and above	0.604	0.33	0.218-1.668	2.09	0.12	0.817-5.352
**Had jaundice**						
Yes	1.			1.0		
No	1.59	0.54	0.363-6.990	1.61	0.59	0.276-9.348
**Had acute or chronic liver dx**						
Yes	1			1.0		
No	1.90	0.54	0.249-14.515	1.30	0.83	0.121-13.935
**Tattoo on body**						
Yes	1			1		
No	1.58	0.66	0.206- 12.107	1.79	0.62	0.179-17.846
**Rejected as blood donor**						
Yes	1			1		
No	0.47	0.48	0.0573-3.834	0.15	0.14	0.012-1.833
**Personal hair treating materials**						
Yes	1			1.0		
No	0.65	0.35	0.258-1.616	0.38	0.09	0.1222-1.170
**Blood transfusion**						
Yes	1			1.0		
No	0.71	0.58	0.235- 2.189	0.41	0.17	0.114-1.469
**Sharing tooth brushes**						
Yes	1			1.0		
No	2.01	0.68	0.264- 15.369	3.70	0.30	0.309-44.359
**Spouse rejected as donor**						
Yes	1			1.0		
No	0.32	0.15	0.0672-1.485	0.21	0.09	0.034-1.256
**HIV status**						
Negative	1					
Positive	0.861	0.88	0.110-6.712	0.79	0.84	0.085-7.429

## Discussion

Our study population was relatively young with a mean age of 30.02 +4.30 years. The majority of respondents had secondary and post secondary education; a finding similar to that reported in a multi-indicator cluster survey of women of child bearing age in Akwa Ibom State 14. More than half of our study population were aware of HCV infection, a finding that is corroborated by the report from a study^15^ which showed that HCV infection is assuming epidemic dimensions, and this is compounded by lack of awareness and knowledge about the infection among at risk populations, health care providers and policy makers.

In this study, the proportion of pregnant women with positive serological markers for HCV was 4.6%. The world-wide prevalence of HCV infection in pregnancy ranges from 0.15-2.4% in the developed world and is estimated to be as high as 8.6% Sub-Saharan Africa^16^. This higher prevalence in this study compared to that of developed countries may be attributed to an increase in blood transfusion rates in pregnant women in developing countries. In this study, the method used was a screening test with possible false positives and no confirmatory diagnostic method was employed. There may also be the risk of selection bias as pregnant women who have risk factors may present to the Teaching hospital, being a referral centre where women with complications would be referred for care from peripheral centres hence inflating the prevalence of the disease condition.

The factors recalled by respondents in this study that could have exposed them to the risk of having HCV infection included sharing of hair making materials, history of blood transfusion, sharing tooth brushes and tattooing which depicts parenteral routes of transmission. These finding agree with findings from other studies that HCV transmission occurs principally through parenteral exposure to contaminated blood or blood products in adults and through mother to child transmission in children with a transmission rate of 6% reported in a study 7. Acupuncture and tattooing and use of unsterilized sharps 5 are other risk factors that have also been reported which still confirms parenteral routes of transmission. History of previous sexually transmitted infections was also elicited in this study. However, HCV is not found consistently in body secretions, thus sexual transmission though possible is rare.^10^ Reports using anti-HCV assays have shown lower rates of HCV transmission from seropositive partners, approximately 0% to 4% 2. A very small percent of respondents in this study reported use of intravenous drugs which however were prescribed intravenous antibiotics in health care settings. Fortunately, intravenous drug use is uncommon in Sub-Saharan Africa.^17^

In this study, there was a statistically significant association between the second trimester of pregnancy and respondents' HCV status both in the univariate and multivariate logistic regression. Respondents in the second trimester were 79% and 82% less likely to be positive for HCV virus infection. Most pregnant women in Nigeria register for antenatal care late, usually in the second trimester^18^ and if found to be anaemic will be given oral hematinics instead of blood transfusion as in the third trimester since there is some time for correction of the anaemia. This way, respondents in second trimester are less likely to be transfused with blood even when found to be anaemic with the reasoning that there is time to correct the anaemia with oral medications. This reduces their parenteral risk of exposure to blood and blood products and protects them from HCV infections. Conversely, considering the short duration before delivery, anaemic pregnant women in the third trimester are more likely to be transfused with blood and hence the increased risk of HCV infection. Apart from these, studies have also shown a corresponding increase in HCV RNA during the 2nd and 3rd trimesters^19^. It is hypothesized that this may be seen because of relative suppression of immunity as pregnancy proceeds^19^. The modulation of cytokines as a result of this is important in maintaining tolerance of the paternal antigens in the foetus, which may also increase the proliferation of HCV resulting in higher HCV RNA titres in the second and third trimesters^20^,^21^. Conversely, HCV RNA titres tend to decrease in the postpartum period.^22^

In addition, increasing gravidity was found to have a statistically significant association with HCV positive status. Every unit increase in gravidity decreased by 62% their risk of HCV infection in the study. This perhaps may be due to increasing awareness among the women with each pregnancy and the practice of prevention of parenteral means of infection from ANC education sessions in health facilities.

The association between respondents with a history of blood transfusion in the past and HCV status was not significant statistically. However, some studies have reported less than ideal universal pre-transfusion screening for HCV and revealed that insufficient screening of transfused blood and blood products and parenteral exposure continue to contribute to HCV transmission in developing countries^17^.

In our study, respondents with previous history of jaundice, chronic liver disease and sharing of tooth brushes were more likely to be HCV positive though this association was not statistically significant. It is also noteworthy that among 69% of the pregnant women who were newly diagnosed, 73% had no identifiable risk factors for hepatitis C. Hence the fact that no risk factors were identified in this study agrees with these studies.

This study was not without some limitations. The results of this study may not be generalizable to a larger population like Uyo metropolis in Akwa Ibom State because the sample consisted of a cohort of pregnant women obtaining ANC in UUTH. This study was cross sectional in nature and determined the perception of respondents and detection of HCV antibodies in participants only at a limited given time. The ABON® Hepatitis C Virus antibody test is limited to the qualitative detection of HCV antibodies in human serum or plasma. If the quantity of antibody present in the serum or plasma is lower than the limit of detection of the test kit, a false negative result can result.

## Conclusion

This study revealed that the prevalence of HCV virus infection among the study population was high. Second trimester and increasing gravidity were protective of HCV infection

## Recommendations

The rising rate of HCV infections, coupled with the availability of highly efficacious treatment regimens for postpartum women and failure of risk-factor based screening to identify most individuals, warrants introduction of general screening of all pregnant women presenting in health facilities to prevent maternal complication and MTCT. In addition, many young women consistently seek care only during pregnancy, and universal screening can easily be packaged within current antenatal screening practices^23^, knowledge of HCV status could influence provider decisions and patients' behaviour as well as help facilitate evaluation of HCV exposed infants^23^.
